# Double edge resolving set and exchange property for nanosheet structure

**DOI:** 10.1016/j.heliyon.2024.e26992

**Published:** 2024-02-28

**Authors:** Ali N.A. Koam, Ali Ahmad, Sikander Ali, Muhammad Kamran Jamil, Muhammad Azeem

**Affiliations:** a Department of Mathematics, College of Science, Jazan University, P.O. Box. 114, Jazan 45142, Kingdom of Saudi Arabia; b Department of Information Technology and Security, College of Computer Science & Information Technology, Jazan University, Jazan, Kingdom of Saudi Arabia; c Department of Mathematics, COMSATS University Islamabad, Sahiwal campus, Pakistan; d Department of Mathematics, Riphah International University, Lahore 54000, Pakistan

**Keywords:** Edge resolving set, Edge metric dimension, Nanosheet, Octogonal grid, Exchange property edge resolving set

## Abstract

The exploration of edge metric dimension and its applications has been an ongoing discussion, particularly in the context of nanosheet graphs formed from the octagonal grid. Edge metric dimension is a concept that involves uniquely identifying the entire edge set of a structure with a selected subset from the vertex set, known as the edge resolving set. Let's consider two distinct edge resolving sets, denoted as $R_{e_{1}}$ and $R_{e_{2}}$, where $R_{e_{1}}\neq R_{e_{2}}$. In such instances, it indicates that the graph *G* possesses a double-edge resolving set. This implies the existence of two different subsets of the vertex set, each capable of uniquely identifying the entire edge set of the graph. In this article, we delve into the edge metric dimension of nanosheet graphs derived from the octagonal grid. Additionally, we initiate a discussion on the exchange property associated with the edge resolving set. The exchange property holds significance in the study of resolving sets, playing a crucial role in comprehending the structure and properties of the underlying graph.

## 1 Introduction

Chemical graph theory is a useful tool for explaining chemical structures and presenting molecular representations mathematically. It is essential for explaining the structural properties of different kinds of materials, including as molecules, polymers, processes, crystals, and clusters. In order to show how mathematical ideas and methods can be used to chemistry, this field of study has expanded to include the most recent advancements and discoveries in mathematical models of chemical conditions [1]. Simplifying and building intricate chemical structures that could be difficult to comprehend in their original form is one noteworthy use of chemical graph theory. Researchers can learn more about the characteristics and actions of various chemical substances by using mathematical representations.

In materials science, octagonal grid-derived nanosheets are crucial and well-researched materials [2], [3], [4]. Because of their thinness, these nanostructures are valued in a variety of sectors, including nanotechnology and medicine. These structures are resolved and their vertex-based metric dimensions are determined [5]. This study advances our knowledge of the special qualities and possible uses of nanosheet chemicals. Further insights and discussions regarding mathematical elements of graph theory and chemistry can be gained from recent works [6], [7]. These papers demonstrate how mathematical chemistry is interdisciplinary and how mathematical models and methods improve our understanding of chemical structures and events.

In graph theory, the idea of an edge-resolving set is essential, especially when discussing chemical structures. A subset of a graph $G^{\prime }s$ vertex set known as an edge resolving set, or $R_{e_{1}}$, is responsible for uniquely identifying each edge in *G* by measuring the distances between the vertices in $R_{e}$ and the edges. This distinctive identification highlights the importance of this idea in chemical graph theory by adding to a distinctive representation of chemical structures [8]. The edge metric dimension is defined as the cardinality of the smallest edge resolving set [9], [10], [11]. This metric gives an indication of how well a collection of vertices resolves the graph's edges. The idea of a locating set, which is a precursor to the metric dimension, was introduced by Slater in 1975 [12] and later termed the metric dimension by Harary in 1976 [13]. The concept of edge metric dimension is independently introduced by Aleksander Kelenc [14]. The exchange property is a significant characteristic associated with resolving sets. It states that for any two minimal resolving sets in a graph, there exists a vertex *u* in $R_{e_{1}}$ and a vertex *v* in $R_{e_{2}}$ such that replacing *u* with *v* in $R_{e_{1}}$ such that $R_{e_{1}}﹨\{u\}\cup v$ results in another minimal resolving set. This exchange property, explored in this study, contributes to the understanding of the set resolution property of graphs [15].

In daily life, the edge metric dimension is used in many ways, which has inspired researchers and has been thoroughly researched. They were specifically employed in pharmaceutical research to identify patterns resembling those of various drugs [16]. Other uses for the edge metric dimension include robot navigation [17], weighing issues, Sonar, coastguard Loran, communications networks [18], image analysis, facility layout issues, and sonar [19], combinatorial optimization [20], [21], and coding and decoding of mastermind games [22]. For more information on the physical and chemical characteristics of the metric dimension, see [23], [24], [25].

Due to the vast range of applications for which it is put, the concept of edge metric dimension is usually used to solve complex problems. Resolving set and exchange property in nanotube discussed in [26]. The metric dimensions of various chemical structures have been studied in numerous articles. Metric dimensions have many applications in chemistry Hollow coronoid's Edge Metric dimension is discussed in [27]. Edge metric dimension and barycentric partition of the Cayley graph were both examined in [28]. In [29], contains groundbreaking work on the edge metric dimension. The structure of curved polytopes is discussed in [30]. Our study can benefit from understanding some fundamental definitions of distance, resolving set, and metric dimension. The concept of metric dimension is applied to resolve a wide range of challenging issues due to its diversity. For variables relating to the resolvability of different chemical graphs, we refer to [31], [32]. For more in-depth exploration of the chemical and physical properties of octagonal grids, please consult the works cited in [33], [34], [35].

Definition 1.1 In a simple, undirected graph G, the distance, also known as a geodesic, between any two vertices is equal to the length of the shortest path in G between those vertices. The symbol for it is *ζ*(${ℵ}_{1},{ℵ}_{2})$. A collection of edges and vertices are represented by E(G) and V(G) respectively.

Definition 1.2 The edge metric dimension of *G*, represented by $dim_{e}$, is the least cardinality of an edge resolving set, operating as an edge metric basis for *G*. Let an ordered set $R_{e}={ℵ}_{1},{ℵ}_{2},{ℵ}_{3},\;dots{ℵ}_{k}$ of the edges of G (vertices of line graph L(G)) and n∈E(G)=V(L(G)), then impression $r(n|R_{e})$ of n about to $R_{e}$ is the k-tuple $\left(d_{e}(n,{ℵ}_{1}),d_{e}(n,{ℵ}_{2})\ldots d_{e}(n,{ℵ}_{k})\right)$, $R_{e}$ is called edge resolving set if $r(n|R_{e})$ is distinct for all n∈E(G).

Theorem 1.3 *Let* $S_{n}$ *be a sunlet graph. Then the edge metric dimension for the sunlet graph is denoted by* $dim_{e}(S_{n})$ *and changes with even and odd values of n have the following edge metric dimension*

$$dim_{e}(S_{n})=\left\{ \begin{array}{c} 2\;\;\text{if}\;\;n\in E\\ 3\;\;\text{if}\;\;n\in O. \end{array}\right.$$

*Here E shows the even number and O is used for the odd number and* $S_{n}$ *is the sunlet graph.*

Theorem 1.4 *Let* $D_{n}$ *be the family of the prism graph. Then* $dim_{e}(D_{n})=3\;\text{if}n\geq 3$ *.*

We have discussed the edge metric dimension of the nanosheet graphs generated from the octagonal grid in this post. We have also spoken about this new structure's exchange characteristics. There is no exchange property stated for such chemical networks or structures in the literature. For the first time, we have attempted to start a conversation on the edge resolving the set's exchange attribute.

## 2 Construction of nanosheet $NS_{h,v}$ derived by octagonal grid

The thickness of the 2D nanostructures is what makes them so significant. They are employed in nanotechnology, nanomedicine, and gene transfer. Their thicknesses range from 1 to 100 nm because of their incredibly thin architectures, nanosheets differ from their bulk counterparts. They are best suited for the administration of various medications along with therapeutic DNA and RNAs due to the high surface-to-volume ratio. Fluid mechanics also use nanosheets. In fluid mechanics, there are numerous papers on nanosheets. Some scholars in the field of graph theory discover topological indices of this nanosheet driven by the octagonal grid, such as the Schultz and Wiener indices by [36], the connective eccentric index, the eccentric connective index, and the eccentric Zagreb index, which was financed by [37]. However, we seek to identify the resolving parameter of this sheet.

**Figure 1:**
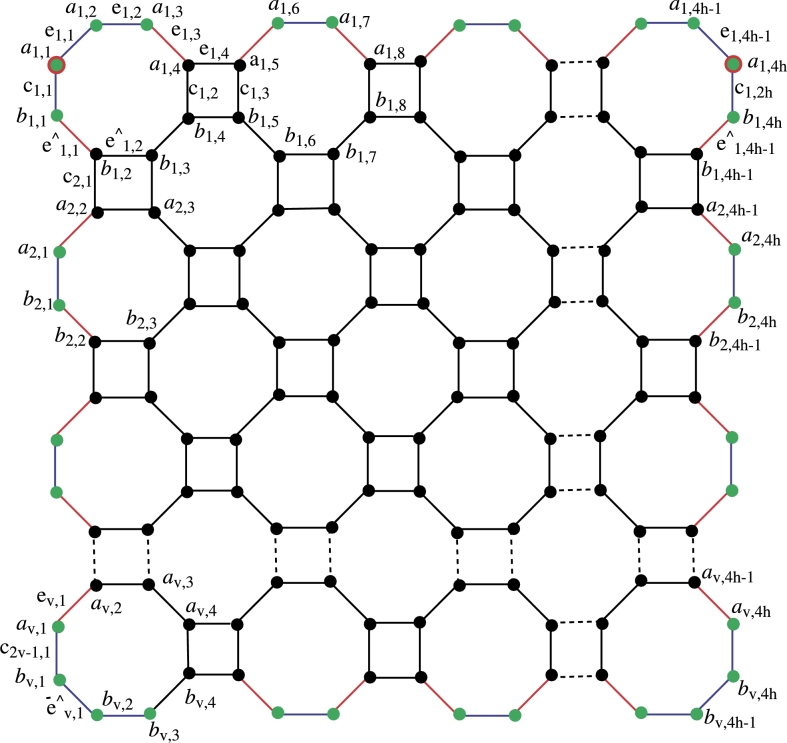
Generalize Nanosheet.

In Fig. 1, the color scheme is as follows: red color is used for edges with endpoints of degree 2 and 3, blue color indicates edges with endpoints of degree 2, and black is assigned to edges with endpoints of degree 3. Green color represents vertices of degree 2, while the black-colored vertex has a degree of 3. Double-colored vertices, such as $a_{1,1}$ and $a_{1,4h}$, are part of the resolving set, marked in green and red due to their degree 2. Let *h* and *v* denote the horizontal and vertical numbers of $C_{8}$, where $h,v\geq 1,\;h,v\in Z^{+}$. The number of vertices with degree 2 is 2hv, and the number of vertices with degree 3 is 8hv−2(hv). The order of $NS_{h,v}$ is $|V(NS_{h,v})|=8hv$, and the size of $NS_{h,v}$ is $\left|E(NS_{h,v})|=12hv-2(h+v\right)$.

In labeling, two parameters, h,v, and two indices, ${ℵ}_{1},{ℵ}_{2}$, are employed. ${ℵ}_{2}$ changes 4 time with *h*, and two time ${ℵ}_{1}$ varies with *v*. In addition, the labeling described above in vertex and edge sets is displayed in Fig. 1 and is used in our primary findings. Furthermore, the margins are labeled as $a_{{ℵ}_{1},{ℵ}_{2}}a_{{ℵ}_{1},{ℵ}_{2}+1}=e_{{ℵ}_{1},{ℵ}_{2}}$, $b_{{ℵ}_{1},{ℵ}_{2}}b_{{ℵ}_{1},{ℵ}_{2}+1}={\overset{ˆ}{e}}_{{ℵ}_{1},{ℵ}_{2}}$ and $a_{{ℵ}_{1},{ℵ}_{2}}b_{{ℵ}_{1},{ℵ}_{2}}=c_{{ℵ}_{1},{ℵ}_{2}}$. The vertex and edge set of the nanosheet is respectively.

$$V(NS)=\{a_{{ℵ}_{1},{ℵ}_{2}},b_{{ℵ}_{1},{ℵ}_{2}};1\leq {ℵ}_{1}\leq v,1\leq {ℵ}_{2}\leq 4h\}E(NS)=\{a_{{ℵ}_{1},{ℵ}_{2}}a_{{ℵ}_{1},{ℵ}_{2}+1},b_{{ℵ}_{1},{ℵ}_{2}}b_{{ℵ}_{1}{ℵ}_{2}+1};1\leq {ℵ}_{1}\leq v,1\leq {ℵ}_{2}\leq 4h\}\cup \{a_{{ℵ}_{1},{ℵ}_{2}}b_{{ℵ}_{1},{ℵ}_{2}};1\leq {ℵ}_{1}\leq v,{ℵ}_{2}=0,1(mod\;4)\}\cup \{a_{{ℵ}_{1},{ℵ}_{2}}b_{{ℵ}_{1},{ℵ}_{2}};1\leq {ℵ}_{1}\leq v,{ℵ}_{2}=2,3(mod\;4)\}.$$

## 3 Double edge resolving sets of nanosheet derived from octagonal grid

Theorem 3.1 *If*$NS_{h,v}$*is an octagonal nanosheet with*h,v≥1*, then double edge resolving sets of cardinality* 2 *exist.*

Proof To prove the double edge resolving set for nanosheet, we define two sets $R_{e_{1}}$ and $R_{e_{2}}$. Now we want to show that $R_{e_{1}}$ and $R_{e_{2}}$ both are edge resolving sets for nanosheet. The prove of $R_{e_{1}}$ is in Theorem 3.2 and prove of $R_{e_{2}}$ present in Theorem 3.3 □

### 3.1 $R_{e_{1}}$ is a resolving set of nanosheet

Theorem 3.2 *If*$NS_{h,v}$*is a nanosheet with*h,v≥1*, then*$R_{e_{1}}$*is a minimal edge-resolving set of cardinality* 2*.*

Proof To prove that $R_{e_{1}}=\{a_{1,1},a_{1,4h}\}$ is a minimal edge-resolving set for $NS_{h,v}$, we will show that $R_{e_{1}}$ is an edge-resolving set. Let *e* be an arbitrary edge in $NS_{h,v}$, and let *d* denote the distance between *e* and the vertices in $R_{e_{1}}$. We will show that this representation is unique. Consider the edges adjacent to the vertices in $R_{e_{1}}$. The vertices $a_{1,1}$ and $a_{1,4h}$ are of degree 2, so they are connected to two edges each. Specifically, $a_{1,1}$ is connected to edges $e_{1,1}$ and $c_{1,1}$, while $a_{1,4h}$ is connected to edges $e_{1,4h}$ and $c_{1,h+1}$. Now, observe the structure of the nanosheet. The vertices $a_{1,1}$ and $a_{1,4h}$ are the only vertices of degree 2, and they are part of the outer cycle of the nanosheet. All other vertices, including those in the inner cycles, have degree 3. Since the nanosheet is constructed in an octagonal pattern, there are no other vertices of degree 2 in the outer cycle. Therefore, any edge *e* must be incident on $a_{1,1}$ or $a_{1,4h}$ or both. Now, consider the distances between the edge *e* and the vertices in $R_{e_{1}}$. Without loss of generality, let *e* be incident on $a_{1,1}$. The distances are as follows: $d(e,a_{1,1})$ is the distance along the cycle containing $a_{1,1}$. $d(e,a_{1,4h})$ is the distance along the outer cycle to $a_{1,4h}$ and then back to $a_{1,1}$. Since the nanosheet is constructed with an octagonal pattern, the distances along these cycles will be distinct. Therefore, the representation $r(e|R_{e_{1}})$ is unique, and $R_{e_{1}}$ is an edge-resolving set. Since $R_{e_{1}}$ is an edge-resolving set, and it has cardinality 2, it is a minimal edge-resolving set for $NS_{h,v}$. The representation for h=1=v present in Table 1

**Table 1:** Edge representation of Fig. 2.

$R_{e_{1}}=\{a_{1,1},a_{1,4}\}$								
*Edges*	*e*_1,1_	*e*_1,2_	*e*_1,3_	${\overset{ˆ}{e}}_{1,1}$	${\overset{ˆ}{e}}_{1,2}$	${\overset{ˆ}{e}}_{1,3}$	*c*_1,1_	*c*_1,2_
$r(.|R_{e_{1}})$	(0,2)	(1,1)	(2,0)	(1,3)	(2,2)	(3,1)	(0,4)	(4,0)

**Generalized Results**

Now, considering the generalized values of *h* and *v* where h,v≥1, the distinct representation of all vertices from $NS_{h,v}$ is presented below. The formulas for distances across all edges in the octagonal nanosheet reveal that the minimal edge resolving set possesses a cardinality of two, as each distance is distinct. Let $d(e_{{ℵ}_{1},{ℵ}_{2}},a_{1,1})=w_{1}$, $d(e_{{ℵ}_{1},{ℵ}_{2}},\;a_{1,4h})=w_{2}$, and $r(e_{{ℵ}_{1},{ℵ}_{2}}|R_{e_{1}})=(w_{1},w_{2})$. Or $r(e_{{ℵ}_{1},{ℵ}_{2}}|R_{e_{1}})=(d(e_{{ℵ}_{1},{ℵ}_{2}},a_{1,1}),d(e_{{ℵ}_{1},{ℵ}_{2}},\;a_{1,4h}))$.

$$w_{1}=\left\{ \begin{array}{cc} 2{ℵ}_{1}+{ℵ}_{2}-3\; & \text{for}\;{ℵ}_{1}=1,2,\;\text{and}\;1\leq {ℵ}_{2}\leq 5,\\ {ℵ}_{1}+{ℵ}_{2}-1\; & \text{for}\;{ℵ}_{1}=2,\;\;1\leq {ℵ}_{2}\leq 5,\\ 4{ℵ}_{1}+{ℵ}_{2}-6\; & \text{for}\;{ℵ}_{1}\geq 2,\;\;{ℵ}_{2}=1,\\ 2{ℵ}_{1}+{ℵ}_{2}-3\; & \text{for}\;1\leq {ℵ}_{1}\leq 2,\\ 2{ℵ}_{1}+{ℵ}_{2}-3\; & \text{for}\;{ℵ}_{2}\geq 4({ℵ}_{1}-1)-2,\;\;{ℵ}_{1}\geq 3,\\ 4{ℵ}_{1}+{ℵ}_{2}-7-z-z_{2}\; & \text{for}\;3\leq {ℵ}_{1}\leq 2,\;{ℵ}_{2}\leq 4{ℵ}_{1}-7, \end{array}\right.$$

where

$$z=\left\{ \begin{array}{c} \left\lfloor \frac{{ℵ}_{2}-1}{4}\right\rfloor \;\text{for}\;\;\;{ℵ}_{2}\leq 4{ℵ}_{1}-7,\\ 0\;\;\;\;\;\;\;\;\;\;\;\;\text{otherwise}, \end{array}\right.z_{1}=\left\{ \begin{array}{c} \left\lfloor \frac{{ℵ}_{2}-2}{4}\right\rfloor \;\text{for}\;{ℵ}_{2}\leq 4{ℵ}_{1}-7,\\ 0\;\;\;\;\;\;\;\;\;\;\;\text{otherwise}, \end{array}\right.$$

$$w_{2}=\left\{ \begin{array}{cc} 4h+2{ℵ}_{1}-{ℵ}_{2}-3\; & \text{for}\;{ℵ}_{1}=1,\;{ℵ}_{2}=4h-3,\\ \;\\ 4h+2{ℵ}_{1}-{ℵ}_{2}-3\; & \text{for}\;{ℵ}_{1}\geq 2,\;{ℵ}_{2}\leq 4h-4{ℵ}_{1}+6,\\ \;\\ 4h+2{ℵ}_{1}-{ℵ}_{2}-2+z_{1}+z_{2}\; & \text{for}\;{ℵ}_{1}\geq 2,\;{ℵ}_{2}\geq 4h-4{ℵ}_{1}+7, \end{array}\right.$$

where

$$z=\left\{ \begin{array}{c} \left\lfloor \frac{4{ℵ}_{1}-4h+{ℵ}_{2}-7}{4}\right\rfloor \;\text{for}\;{ℵ}_{1}\geq 2,\;{ℵ}_{2}\leq 4h-4{ℵ}_{1}+6,\\ 0\;\;\;\;\;\;\;\;\;\;\;\text{otherwise}, \end{array}\right.$$

$$z_{1}=\left\{ \begin{array}{c} \left\lfloor \frac{4{ℵ}_{1}-4h+{ℵ}_{2}-6}{2}\right\rfloor \;\text{for}\;{ℵ}_{1}\geq 2,\;{ℵ}_{2}\leq 4h-4{ℵ}_{1}+6,\\ 0\;\;\;\;\;\;\;\;\;\;\;\text{otherwise}, \end{array}\right.$$

$$z_{2}=\left\{ \begin{array}{c} \left\lfloor \frac{{ℵ}_{1}-1}{2}\right\rfloor \;\text{for}\;{ℵ}_{2}\geq {ℵ}_{1}+2,\;{ℵ}_{2}=2E+1\;where\;E\in Z^{+},\\ 0\;\;\;\;\;\;\;\;\;\;\;\;\text{otherwise}. \end{array}\right.$$

So the formula $r(e_{{ℵ}_{1},{ℵ}_{2}}|R_{e_{1}})=(d(e_{{ℵ}_{1},{ℵ}_{2}},a_{1,1}),d(e_{{ℵ}_{1},{ℵ}_{2}},\;a_{1,4h}))$ explains the uniques representations of $e_{{ℵ}_{1},{ℵ}_{2}}$ edges with respect to the *R*. Let $d(c_{{ℵ}_{1},{ℵ}_{2}},a_{1,1})=w_{3}$, $d(c_{{ℵ}_{1},{ℵ}_{2}},a_{1,4h})=w_{4}$, and $r(c_{{ℵ}_{1},{ℵ}_{2}}|R_{e_{1}})=(w_{3},w_{4})$

$$w_{3}=\left\{ \begin{array}{cc} 2{ℵ}_{1}+{ℵ}_{2}-3\; & \text{for}\;{ℵ}_{1}\geq 1,\;{ℵ}_{2}\leq {ℵ}_{1},\\ \;\\ 2{ℵ}_{1}+{ℵ}_{2}-1+z+z_{1}+z_{2}\; & \text{for}\;{ℵ}_{1}\geq 1,\;{ℵ}_{2}\geq {ℵ}_{1}+1, \end{array}\right.$$

where

$$z=\left\{ \begin{array}{c} \left\lfloor \frac{{ℵ}_{1}-2}{2}\right\rfloor \;\text{for}\;{ℵ}_{2}\geq {ℵ}_{1},\\ 0\;\;\;\;\;\;\;\;\;\;\;\text{otherwise}, \end{array}\right.z_{1}=\left\{ \begin{array}{c} \left\lfloor \frac{{ℵ}_{1}}{2}\right\rfloor \;\text{for}\;{ℵ}_{2}\geq {ℵ}_{1}+1,\;{ℵ}_{2}=2E,\\ 0\;\;\;\;\;\;\;\;\;\;\;\text{otherwise}, \end{array}\right.$$

$$z_{2}=\left\{ \begin{array}{c} \left\lfloor \frac{{ℵ}_{1}-1}{2}\right\rfloor \;\;\;{ℵ}_{2}\geq {ℵ}_{1}+2,\;{ℵ}_{2}=2E+1\;where\;E\in Z^{+},\\ 0\;\;\;\;\;\;\;\;\;\;\;\;\text{otherwise}. \end{array}\right.$$

$$w_{4}=\left\{ \begin{array}{cc} 2h+2{ℵ}_{1}-{ℵ}_{2}-2\; & \text{for}\;{ℵ}_{1}\geq 1,\;{ℵ}_{2}\geq 2h-{ℵ}_{1}+1,\\ \;\\ 4h+2{ℵ}_{1}-{ℵ}_{2}-2-2z-z_{1}-z_{2}\; & \text{for}\;{ℵ}_{1}\geq 1,\;\;1\leq {ℵ}_{2}\leq 2h-{ℵ}_{1}, \end{array}\right.$$

where

$$z=\left\{ \begin{array}{c} \left\lfloor \frac{{ℵ}_{2}}{2}\right\rfloor \;\text{for}\;{ℵ}_{2}\geq 1,\\ 0\;\;\;\;\;\;\;\;\;\;\;\text{otherwise}, \end{array}\right.z_{1}=\left\{ \begin{array}{c} \left\lfloor \frac{{ℵ}_{1}}{2}\right\rfloor \;\text{for}\;{ℵ}_{2}\geq 1,\;{ℵ}_{2}=2E+1,\\ 0\;\;\;\;\;\;\;\;\;\;\;\text{otherwise}, \end{array}\right.$$

$$z_{2}=\left\{ \begin{array}{c} \left\lfloor \frac{{ℵ}_{1}-1}{2}\right\rfloor \;\text{for}\;{ℵ}_{2}\geq 1,\;{ℵ}_{2}=2E\;where\;E\in Z^{+},\\ 0\;\;\;\;\;\;\;\;\;\;\;\;\text{otherwise}. \end{array}\right.$$

Let $d({\overset{ˆ}{e}}_{{ℵ}_{1},{ℵ}_{2}},a_{1,1})=w_{1}^{\prime }$, $d({\overset{ˆ}{e}}_{{ℵ}_{1},{ℵ}_{2}},a_{1,4h})=w_{2}^{\prime }$, and $r({\overset{ˆ}{e}}_{{ℵ}_{1},{ℵ}_{2}}|R_{e_{1}})=(w_{1}^{\prime },w_{2}^{\prime })$

$$w_{1}^{\prime }=\left\{ \begin{array}{cc} 2{ℵ}_{1}+{ℵ}_{2}-2\; & \text{for}\;{ℵ}_{1}\geq 1,\;{ℵ}_{2}\geq 4{ℵ}_{1}-4,\\ \;\\ 4{ℵ}_{1}+{ℵ}_{2}-4-z_{1}-z_{2}\; & \text{for}\;{ℵ}_{1}\geq 1,\;\;{ℵ}_{2}\leq 4{ℵ}_{1}-5, \end{array}\right.$$

where

$$z=\left\{ \begin{array}{c} \left\lfloor \frac{{ℵ}_{2}+1}{4}\right\rfloor \;\text{for}\;\;\;{ℵ}_{2}\leq 4{ℵ}_{1}-5,\\ 0\;\;\;\;\;\;\;\;\;\;\;\;\text{otherwise}, \end{array}\right.z_{1}=\left\{ \begin{array}{c} \left\lfloor \frac{{ℵ}_{2}}{4}\right\rfloor \;\text{for}\;{ℵ}_{2}\leq 4{ℵ}_{1}-5,\\ 0\;\;\;\;\;\;\;\;\;\;\;\text{otherwise}, \end{array}\right.$$

$$w_{2}^{\prime }=\left\{ \begin{array}{cc} 4h+2{ℵ}_{1}-{ℵ}_{2}-2\; & \text{for}\;{ℵ}_{1}\geq 1,\;{ℵ}_{2}\leq 4h-4{ℵ}_{1}+4,\\ \;\\ 2h+2{ℵ}_{1}-{ℵ}_{2}-1+z+z_{1}\; & \text{for}\;{ℵ}_{1}\geq 2,\;{ℵ}_{2}\geq 4h-4{ℵ}_{1}-3, \end{array}\right.$$

where

$$z=\left\{ \begin{array}{c} \left\lfloor \frac{4{ℵ}_{1}-4h+{ℵ}_{2}-2}{4}\right\rfloor \;\text{for}\;{ℵ}_{2}\geq 4h-4{ℵ}_{1}-3,\\ 0\;\;\;\;\;\;\;\;\;\;\;\text{otherwise}, \end{array}\right.z_{1}=\left\{ \begin{array}{c} \left\lfloor \frac{4{ℵ}_{1}-4h+{ℵ}_{2}-1}{4}\right\rfloor \;\text{for}\;{ℵ}_{2}\geq 4h-4{ℵ}_{1}-3,\\ 0\;\;\;\;\;\;\;\;\;\;\;\text{otherwise}, \end{array}\right.$$

$$z_{2}=\left\{ \begin{array}{c} \left\lfloor \frac{{ℵ}_{1}-1}{2}\right\rfloor \;\;\;{ℵ}_{2}\geq {ℵ}_{1}+2,\;{ℵ}_{2}=2E+1\;where\;E\in Z^{+},\\ 0\;\;\;\;\;\;\;\;\;\;\;\;\text{otherwise}. \end{array}\right.$$

Let $\zeta_{1}$ and $\zeta_{2}$ be two any arbitrary vertices on nanosheet $NS_{h,v}$, and *d* use for distance. Suppose WLOG represents without loss of generality and let $R=\{a_{1,1},a_{1,4h}\}$

**Case I:** When $\zeta_{1}=e_{{ℵ}_{1},{ℵ}_{2}}$ and $\zeta_{2}=e_{{ℵ}_{1}^{\prime },{ℵ}_{2}^{\prime }}$ then further three cases arise.

**subcase1:** If ${ℵ}_{1}={ℵ}_{1}^{\prime }$, ${ℵ}_{2}\neq {ℵ}_{2}^{\prime }$ then WLOG say that ${ℵ}_{2}<{ℵ}_{2}^{\prime }$ then $d(\zeta_{1},a_{1,1})\neq d(\zeta_{2},a_{1,1})$ because $d(\zeta_{1},a_{1,1})=d(\zeta_{2},a_{1,1})+t$ where $t={ℵ}_{2}^{\prime }-{ℵ}_{2}$ so $r(\zeta_{1}|R_{e_{1}})\neq r(\zeta_{2}|R_{e_{1}})$.

**subcase2:** If ${ℵ}_{1}\neq {ℵ}_{1}^{\prime }$, ${ℵ}_{2}={ℵ}_{2}^{\prime }$ then WLOG say that ${ℵ}_{1}<{ℵ}_{1}^{\prime }$ then $d(\zeta_{1},a_{1,1})\neq d(\zeta_{2},a_{1,1})$ because $d(\zeta_{1},a_{1,1})=d(\zeta_{2},a_{1,1})+s$ where $s=2({ℵ}_{1}^{\prime }-{ℵ}_{1})$ so $r(\zeta_{1}|R_{e_{1}})\neq r(\zeta_{2}|R_{e_{1}})$.

**subcase3:** If ${ℵ}_{1}\neq {ℵ}_{1}^{\prime }$, ${ℵ}_{2}\neq {ℵ}_{2}^{\prime }$ then WLOG say that ${ℵ}_{2}<{ℵ}_{2}^{\prime }$, ${ℵ}_{1}<{ℵ}_{1}^{\prime }$ then $d(\zeta_{1},R_{e_{1}})\neq d(\zeta_{2},R_{e_{1}})$ because $d(\zeta_{1},R_{e_{1}})=d(\zeta_{2},R_{e_{1}})+(s+t)$ so $r(\zeta_{1}|R_{e_{1}})\neq r(\zeta_{2}|R_{e_{1}})$.

**Case II:** When $\zeta_{1}={\overset{ˆ}{e}}_{{ℵ}_{1},{ℵ}_{2}}$ and $\zeta_{2}={\overset{ˆ}{e}}_{{ℵ}_{1}^{\prime },{ℵ}_{2}^{\prime }}$ then also three cases more.

**subcase1:** If ${ℵ}_{1}={ℵ}_{1}^{\prime }$, ${ℵ}_{2}\neq {ℵ}_{2}^{\prime }$ then WLOG say that ${ℵ}_{2}<{ℵ}_{2}^{\prime }$ then $d(\zeta_{1},a_{1,1})\neq d(\zeta_{2},a_{1,1})$ because $d(\zeta_{1},a_{1,1})=d(\zeta_{2},a_{1,1})+t$ where $t={ℵ}_{2}^{\prime }-{ℵ}_{2}$ so $r(\zeta_{1}|R_{e_{1}})\neq r(\zeta_{2}|R_{e_{1}})$.

**subcase2:** If ${ℵ}_{1}\neq {ℵ}_{1}^{\prime }$, ${ℵ}_{2}={ℵ}_{2}^{\prime }$ then WLOG say that ${ℵ}_{1}<{ℵ}_{1}^{\prime }$ then $d(\zeta_{1},a_{1,1})\neq d(\zeta_{2},a_{1,1})$ because $d(\zeta_{1},a_{1,1})=d(\zeta_{2},a_{1,1})+s$ where $s=2({ℵ}_{1}^{\prime }-{ℵ}_{1})$ so $r(\zeta_{1}|R_{e_{1}})\neq r(\zeta_{2}|R_{e_{1}})$.

**subcase3:** If ${ℵ}_{1}\neq {ℵ}_{1}^{\prime }$, ${ℵ}_{2}\neq {ℵ}_{2}^{\prime }$ then WLOG say that ${ℵ}_{2}<{ℵ}_{2}^{\prime }$, ${ℵ}_{1}<{ℵ}_{1}^{\prime }$ then $d(\zeta_{1},R_{e_{1}})\neq d(\zeta_{2},R_{e_{1}})$ because $d(\zeta_{1},R_{e_{1}})=d(\zeta_{2},R_{e_{1}})+(s+t)$ so $r(\zeta_{1}|R_{e_{1}})\neq r(\zeta_{2}|R_{e_{1}})$.

**Case III:** When $\zeta_{1}=e_{{ℵ}_{1},{ℵ}_{2}}$ and $\zeta_{2}={\overset{ˆ}{e}}_{{ℵ}_{1}^{\prime },{ℵ}_{2}^{\prime }}$ then further four cases arise.

**subcase1:** If ${ℵ}_{1}={ℵ}_{1}^{\prime }$, ${ℵ}_{2}={ℵ}_{2}^{\prime }$ then $d(\zeta_{1},a_{1,1})\neq d(\zeta_{2},a_{1,1})$ because $d(\zeta_{1},a_{1,1})$ least is comparable to $d(\zeta_{2},a_{1,1})+1$ so $r(\zeta_{1}|R_{e_{1}})\neq r(\zeta_{2}|R_{e_{1}})$.

**subcase2:** If ${ℵ}_{1}={ℵ}_{1}^{\prime }$, ${ℵ}_{2}\neq {ℵ}_{2}^{\prime }$ then WLOG say that ${ℵ}_{2}<{ℵ}_{2}^{\prime }$ then $d(\zeta_{1},a_{1,1})\neq d(\zeta_{2},a_{1,1})$ because $d(\zeta_{1},a_{1,1})$ least is comparable to $d(\zeta_{2},a_{1,1})+2$ so $r(\zeta_{1}|R_{e_{1}})\neq r(\zeta_{2}|R_{e_{1}})$.

**subcase3:** If ${ℵ}_{1}\neq {ℵ}_{1}^{\prime }$, ${ℵ}_{2}={ℵ}_{2}^{\prime }$ then WLOG say that ${ℵ}_{1}<{ℵ}_{1}^{\prime }$ then $d(\zeta_{1},a_{1,1})\neq d(\zeta_{2},a_{1,1})$ because $d(\zeta_{1},a_{1,1})$ least is comparable to $d(\zeta_{2},a_{1,1})+3$ so $r(\zeta_{1}|R_{e_{1}})\neq r(\zeta_{2}|R_{e_{1}})$.

**Case 4:** If ${ℵ}_{1}\neq {ℵ}_{1}^{\prime }$, ${ℵ}_{2}\neq {ℵ}_{2}^{\prime }$ then WLOG say that ${ℵ}_{2}<{ℵ}_{2}^{\prime }$, ${ℵ}_{1}<{ℵ}_{1}^{\prime }$ then $d(\zeta_{1},a_{1,1})\neq d(\zeta_{2},a_{1,1})$ because $d(\zeta_{1},a_{1,1})$ least is comparable to $d(\zeta_{2},a_{1,1})+2$ so $r(\zeta_{1}|R_{e_{1}})\neq r(\zeta_{2}|R_{e_{1}})$.

When ${ℵ}_{1}\neq {ℵ}_{1}^{\prime }$, ${ℵ}_{2}\neq {ℵ}_{2}^{\prime }$ the positions of ${ℵ}_{1}$ and ${ℵ}_{2}$ where $d(\zeta_{1},a_{1,1})=d(\zeta_{2},a_{1,1})$ then $d(\zeta_{1},a_{1,4h})\neq d(\zeta_{2},a_{1,4h})$.

**Figure 2:**
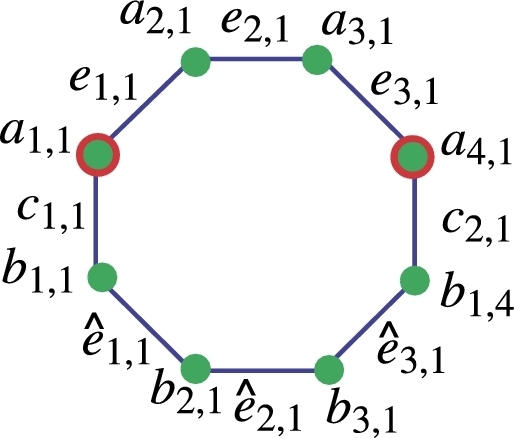
Octagone for sheet.

One can note from the Fig. 2 when $d(\zeta_{1},a_{1,1})=d(\zeta_{2},a_{1,1})$ then $d(\zeta_{2},a_{1,4h})\neq d(\zeta_{1},a_{1,4h})$ also $d(\zeta_{2},a_{1,4h})=d(\zeta_{1},a_{1,4h})$ then $d(\zeta_{1},a_{1,1})\neq d(\zeta_{2},a_{1,1})$.

we discuss all cases where $d(\zeta_{1},a_{1,1})\neq d(\zeta_{2},a_{1,1})$ while $d(\zeta_{1},a_{1,4h})=d(\zeta_{2},a_{1,4h})$

or $d(\zeta_{1},a_{1,1})=d(\zeta_{2},a_{1,1})$, $d(\zeta_{1},a_{1,4h})\neq d(\zeta_{2},a_{1,4h})$ No, any possibility showing two representations are the same. So $R_{e_{1}}$ has cardinality 2.

**Contrary Case**

In all the above discussion we have proven that the cardinality of the resolving set is less or equal to 2. Now we want to show that the cardinality of the resolving set is not greater than 2.

$$minimal\;of\;Cardinality\;of\;\;edge\;resolving\;set\;of\;NS_{h,v}\geq 2∼\;minimal\;of\;cardinality\;of\;edge\;resolving\;set\;of\;NS_{h,v}<2\Rightarrow \;minimal\;of\;cardinality\;of\;minimal\;edge\;resolving\;set\;of\;NS_{h,v}=1$$

contradiction the edge resolving set of $N_{h,v}$ is possible if and only if it is a path graph, and also it contains cycle and the minimum edge metric dimension 2 if a graph contains a cycle so the minimal edge resolving a set of cardinality 1 is not possible.

→ the minimal edge resolving set of $NS_{h,v}\geq 1$. Hence prove that the minimal edge resolving set of $N_{h,v}$ has cardinality 2. □

### 3.2 $R_{e_{2}}$ is also edge resolving set of nanosheet

In this section, we discuss the other edge resolving set $R_{e_{2}}$ of nanosheet derived by octagonal grid.

#### 3.2.1 Construction of nanosheet $NS_{h,v}$ derived by octagonal grid

**Figure 3:**
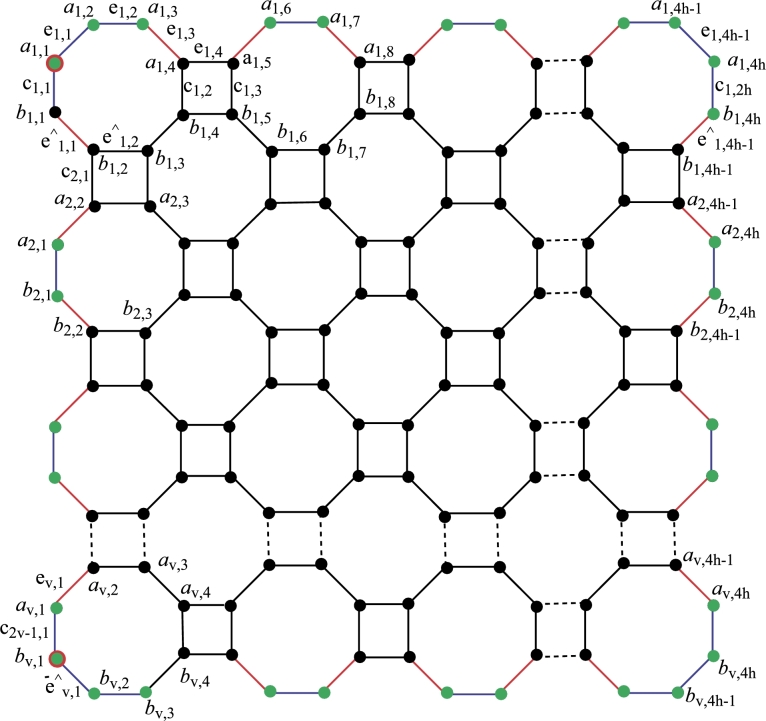
Generalize Nanosheet.

Red color is used in Fig. 3 to indicate edges with endpoints of degrees 2 and 3. For edges with degree 2 endpoints, the color is blue, and for edges with degree 3 endpoints, the color is black. For vertices of degree two, the color green is utilized, and for vertices of degree three, the color black. The vertices with double colors, such as $a_{1,1}$ and $b_{v,1}$, are crucial points in the resolving set. These vertices are marked with green and red colors, indicating a degree of 2, and they are essential members of the resolving set. Let *h* and *v* represent the horizontal and vertical numbers in the context of $C_{8}$, where $h,v\geq 1,\;h,v\in Z^{+}$. The count of vertices with a degree of 2 is 4(h+v), and the count of nodes with a degree of 3 is 8hv−2(hv). The order of $NS_{h,v}$ is given by $|V(NS_{h,v})|=8hv$, and the size of $NS_{h,v}$ is yet to be determined. $\left|E(NS_{h,v})|=12hv-2(h+v\right)$. The edges are labeled as $a_{{ℵ}_{1},{ℵ}_{2}}a_{{ℵ}_{1},{ℵ}_{2}+1}=e_{{ℵ}_{1},{ℵ}_{2}}$, $b_{{ℵ}_{1},{ℵ}_{2}}b_{{ℵ}_{1},{ℵ}_{2}+1}={\overset{ˆ}{e}}_{{ℵ}_{1},{ℵ}_{2}}$ and $a_{{ℵ}_{1},{ℵ}_{2}}b_{{ℵ}_{1},{ℵ}_{2}}=c_{{ℵ}_{1},{ℵ}_{2}}$. The size and order of the graph are the same as the above graph.

Theorem 3.3 *Let*$N_{h,v}$*be a nanosheet with*h,v≥1*then an other edge resolving set*$R_{e_{2}}$*of cardinality* 2 *exist.*

Proof To prove that the other edge resolving set $R_{e_{2}}$ of $N_{h,v}$ has minimum cardinality is also 2. Now to prove this claim we will follow the definition of edge resolving set. The edge resolving set is defined as $R_{e_{2}}=\{a_{1,1},b_{v,1}\}$. Given below are the unique representation of all edges of $NS_{h,v}$ for h,v≥1. The representation for h=1=v present in Table 2

**Table 2:** Edge representation of Fig. 4.

$R_{e_{2}}=\{b_{1,1},b_{1,4}\}$								
*Edges*	*e*_1,1_	*e*_1,2_	*e*_1,3_	${\overset{ˆ}{e}}_{1,1}$	${\overset{ˆ}{e}}_{1,2}$	${\overset{ˆ}{e}}_{1,3}$	*c*_1,1_	*c*_1,2_
$r(.|R_{e_{2}})$	(0,1)	(1,2)	(2,3)	(1,0)	(2,1)	(3,2)	(0,0)	(3,3)

**Generalized Results**

Now, considering the generalized values of *h* and *v* where h,v≥1, the distinct representation of all vertices from $N_{h,v}$ is presented below. The formulas for distances across all edges in the octagonal nanosheet reveal that the minimal edge resolving set possesses a cardinality of two, as each distance is distinct. Let $d(e_{{ℵ}_{1},{ℵ}_{2}},a_{1,1})=w_{1}$, $d(e_{{ℵ}_{1},{ℵ}_{2}},a_{1,4h})=w_{2}$, and $r(e_{{ℵ}_{1},{ℵ}_{2}}|R_{e_{2}})=(w_{1},w_{2})$

$$w_{1}=\left\{ \begin{array}{cc} 2{ℵ}_{1}+{ℵ}_{2}-3\; & \text{for}\;{ℵ}_{1}=1,2,\;\text{and}\;1\leq {ℵ}_{2}\leq 5,\\ {ℵ}_{1}+{ℵ}_{2}-1\; & \text{for}\;{ℵ}_{1}=2,\;\;1\leq {ℵ}_{2}\leq 5,\\ 4({ℵ}_{1}+{ℵ}_{2}-6\; & \text{for}\;{ℵ}_{1}\geq 2,\;\;{ℵ}_{2}=1,\\ 2{ℵ}_{1}+{ℵ}_{2}-3\; & \text{for}\;1\leq {ℵ}_{1}\leq 2,\\ 2{ℵ}_{1}+{ℵ}_{2}-3\; & \text{for}\;{ℵ}_{2}\geq 4({ℵ}_{1}-1)-2,\;\;{ℵ}_{1}\geq 3,\\ 4{ℵ}_{1}+{ℵ}_{2}-7-z-z_{2}\; & \text{for}\;3\leq {ℵ}_{1}\leq 2,\;{ℵ}_{2}\leq 4{ℵ}_{1}-7, \end{array}\right.$$

where

$$z=\left\{ \begin{array}{c} \left\lfloor \frac{{ℵ}_{2}-1}{4}\right\rfloor \;\text{for}\;\;\;{ℵ}_{2}\leq 4{ℵ}_{1}-7,\\ 0\;\;\;\;\;\;\;\;\;\;\;\;\text{otherwise}, \end{array}\right.z_{1}=\left\{ \begin{array}{c} \left\lfloor \frac{{ℵ}_{2}-2}{4}\right\rfloor \;\text{for}\;{ℵ}_{2}\leq 4{ℵ}_{1}-7,\\ 0\;\;\;\;\;\;\;\;\;\;\;\text{otherwise}, \end{array}\right.$$

$$W_{2}=\left\{ \begin{array}{cc} 4v-4{ℵ}_{1}+{ℵ}_{2}-z-z_{1}\; & \text{for}\;1\leq {ℵ}_{1}\leq v\;\text{and}\;1\leq {ℵ}_{2}\leq 4h-4{ℵ}_{1},\\ 2v-2{ℵ}_{1}+{ℵ}_{2}\; & \text{for}\;1\leq {ℵ}_{1}\leq v,\;4v-4{ℵ}_{1}\leq {ℵ}_{2}\leq 4h, \end{array}\right.$$

where

$$z=\left\{ \begin{array}{c} \left\lfloor \frac{{ℵ}_{2}+1}{4}\right\rfloor \;\text{for}\;\;\;\;1\leq {ℵ}_{2}\leq 4h-4{ℵ}_{1},\\ 0\;\;\;\;\;\;\;\;\;\;\;\;\;\text{otherwise}, \end{array}\right.z_{1}=\left\{ \begin{array}{c} \left\lfloor \frac{{ℵ}_{2}}{4}\right\rfloor \;\text{for}\;\;\;\;1\leq {ℵ}_{2}\leq 4h-4{ℵ}_{1},\\ 0\;\;\;\;\;\;\;\;\;\;\;\;\;\text{otherwise}. \end{array}\right.$$

Let $d({\overset{ˆ}{e}}_{{ℵ}_{1},{ℵ}_{2}},a_{1,1})=w_{1}^{\prime }$, $d({\overset{ˆ}{e}}_{{ℵ}_{1},{ℵ}_{2}},a_{1,4h})=w_{2}^{\prime }$, and $r({\overset{ˆ}{e}}_{{ℵ}_{1},{ℵ}_{2}}|R_{e_{2}})=(w_{1}^{\prime },w_{2}^{\prime })$

$$w_{1}^{\prime }=\left\{ \begin{array}{cc} 2{ℵ}_{1}+{ℵ}_{2}-2\; & \text{for}\;{ℵ}_{1}\geq 1,\;{ℵ}_{2}\geq 4{ℵ}_{1}-4,\\ \;\\ 4{ℵ}_{1}+{ℵ}_{2}-4-z_{1}-z_{2}\; & \text{for}\;{ℵ}_{1}\geq 1,\;\;{ℵ}_{2}\leq 4{ℵ}_{1}-9, \end{array}\right.$$

where

$$z=\left\{ \begin{array}{c} \left\lfloor \frac{{ℵ}_{2}+1}{4}\right\rfloor \;\text{for}\;\;\;{ℵ}_{2}\leq 4{ℵ}_{1}-5,\\ 0\;\;\;\;\;\;\;\;\;\;\;\;\text{otherwise}, \end{array}\right.z_{1}=\left\{ \begin{array}{c} \left\lfloor \frac{{ℵ}_{2}}{4}\right\rfloor \;\text{for}\;{ℵ}_{2}\leq 4{ℵ}_{1}-5,\\ 0\;\;\;\;\;\;\;\;\;\;\;\text{otherwise}, \end{array}\right.$$

$$W_{2}^{\prime }=\left\{ \begin{array}{cc} 4v-4{ℵ}_{1}+{ℵ}_{2}-z_{2}-z_{3}\; & \text{for}\;1\leq {ℵ}_{1}\leq v-1,\;\;1\leq {ℵ}_{2}\leq 4h-4{ℵ}_{1}-6,\\ \;\\ 4v-4{ℵ}_{1}+{ℵ}_{2}-3-2z_{3}\; & \text{for}\;1\leq {ℵ}_{1}\leq v,\;\;1\leq {ℵ}_{2}\leq 4h-4{ℵ}_{1}-3,\\ 2v-2{ℵ}_{1}+{ℵ}_{2}-1\; & \text{for}\;1\leq {ℵ}_{1}\leq v,\;\;4v-4{ℵ}_{1}-7\leq {ℵ}_{2}\leq 4h, \end{array}\right.$$

where

$$z_{3}=\left\{ \begin{array}{c} \left\lfloor \frac{{ℵ}_{2}-1}{4}\right\rfloor \;\text{for}\;1\leq 4{ℵ}_{2}\leq 4h-4{ℵ}_{1}-6,\\ 0\;\;\;\;\;\;\;\;\;\;\;\;\text{otherwise}, \end{array}\right.z_{4}=\left\{ \begin{array}{c} \left\lfloor \frac{{ℵ}_{2}+2}{4}\right\rfloor \;\text{for}\;1\leq 4{ℵ}_{2}\leq 4h-4{ℵ}_{1}-6,\\ 0\;\;\;\;\;\;\;\;\;\;\;\;\text{otherwise}. \end{array}\right.$$

Let $d(c_{{ℵ}_{1},{ℵ}_{2}},a_{1,1})=w_{3}$, $d(c_{{ℵ}_{1},{ℵ}_{2}},a_{1,4h})=w_{4}$, and $r(c_{{ℵ}_{1},{ℵ}_{2}}|R_{e_{2}})=(w_{3},w_{4})$

$$w_{3}=\left\{ \begin{array}{cc} 2{ℵ}_{1}+{ℵ}_{2}-3\; & \text{for}\;{ℵ}_{1}\geq 1,\;{ℵ}_{2}\leq {ℵ}_{1},\\ \;\\ 2{ℵ}_{1}+{ℵ}_{2}-1+z+z_{1}+z_{2}\; & \text{for}\;{ℵ}_{1}\geq 1,\;{ℵ}_{2}\geq {ℵ}_{1}+1, \end{array}\right.$$

where

$$z=\left\{ \begin{array}{c} \left\lfloor \frac{{ℵ}_{2}-2}{2}\right\rfloor \;\text{for}\;{ℵ}_{2}\geq {ℵ}_{1},\\ 0\;\;\;\;\;\;\;\;\;\;\;\text{otherwise}, \end{array}\right.z_{1}=\left\{ \begin{array}{c} \left\lfloor \frac{{ℵ}_{1}}{2}\right\rfloor \;\text{for}\;{ℵ}_{2}\geq {ℵ}_{1}+1,\;{ℵ}_{2}=2E,\\ 0\;\;\;\;\;\;\;\;\;\;\;\text{otherwise}, \end{array}\right.$$

$$z_{2}=\left\{ \begin{array}{c} \left\lfloor \frac{{ℵ}_{1}-1}{2}\right\rfloor \;\;\;{ℵ}_{2}\geq {ℵ}_{1}+2,\;{ℵ}_{2}=2E+1\;where\;E\in Z^{+},\\ 0\;\;\;\;\;\;\;\;\;\;\;\;\text{otherwise}. \end{array}\right.$$

$$w_{4}=\left\{ \begin{array}{cc} 2v-2{ℵ}_{1}+{ℵ}_{2}+9\; & \text{for}\;1\leq {ℵ}_{1}\leq v,\;1\leq {ℵ}_{2}\geq 2h-{ℵ}_{1},\\ \;\\ 4v-2{ℵ}_{1}+{ℵ}_{2}-1-2z_{3}\; & \text{for}\;1\leq {ℵ}_{1}\leq v,\;\;2h-{ℵ}_{1}+1\leq {ℵ}_{2}\leq 2h, \end{array}\right.$$

where

$$z_{3}=\left\{ \begin{array}{c} \left\lfloor \frac{{ℵ}_{2}-2h+{ℵ}_{1}-1}{2}\right\rfloor \;\text{for}\;{ℵ}_{2}\geq 1,\\ 0\;\;\;\;\;\;\;\;\;\;\;\text{otherwise}. \end{array}\right.$$

Let $\zeta_{1}$ and $\zeta_{2}$ be two any arbitrary vertices on nanosheet $NS_{h,v}$. Let $R_{e_{2}}=\{a_{1,1},b_{v,1}\}$

**Case I:** When $\zeta_{1}=a_{{ℵ}_{1},{ℵ}_{2}}$ and $\zeta_{2}=a_{{ℵ}_{1}^{\prime },{ℵ}_{2}^{\prime }}$ then three more Subcases emerge.

**Subcase 1:** If ${ℵ}_{1}={ℵ}_{1}^{\prime }$, ${ℵ}_{2}\neq {ℵ}_{2}^{\prime }$ then WLOG (w.l.o.g) ${ℵ}_{2}<{ℵ}_{2}^{\prime }$ implication would be that $d(\zeta_{1},a_{1,1})\neq d(\zeta_{2},a_{1,1})$, because $d(\zeta_{1},a_{1,1})=d(\zeta_{2},a_{1,1})+t$ where $t={ℵ}_{2}^{\prime }-{ℵ}_{2}$ so $r(\zeta_{1}|R_{e_{2}})\neq r(\zeta_{2}|R_{e_{2}})$.

**Subcase 2:** If ${ℵ}_{1}\neq {ℵ}_{1}^{\prime }$, ${ℵ}_{2}={ℵ}_{2}^{\prime }$ then WLOG say ${ℵ}_{1}<{ℵ}_{1}^{\prime }$ implication would be that $d(\zeta_{1},a_{1,1})\neq d(b,a_{1,1})$, because $d(\zeta_{1},a_{1,1})=d(\zeta_{2},a_{1,1})+s$ where $s=2({ℵ}_{1}^{\prime }-{ℵ}_{1})$ so $r(\zeta_{1}|R_{e_{2}})\neq r(\zeta_{2}|R_{e_{2}})$.

**Subcase 3:** If ${ℵ}_{1}\neq {ℵ}_{1}^{\prime }$, ${ℵ}_{2}\neq {ℵ}_{2}^{\prime }$ then WLOG say ${ℵ}_{2}<{ℵ}_{2}^{\prime }$, ${ℵ}_{1}<{ℵ}_{1}^{\prime }$ implication would be that $d(\zeta_{1},R_{e_{2}})\neq d(\zeta_{2},R_{e_{1}})$, because $d(\zeta_{1},R_{e_{2}})=d(\zeta_{2},R_{e_{2}})+(s+t)$ so $r(\zeta_{1}|R_{e_{2}})\neq r(\zeta_{2}|R_{e_{2}})$.

**Case II:** When $\zeta_{1}=b_{{ℵ}_{1},{ℵ}_{2}}$ and $\zeta_{2}=b_{{ℵ}_{1}^{\prime },{ℵ}_{2}^{\prime }}$ then also three Subcases more.

**Subcase 1:** If ${ℵ}_{1}={ℵ}_{1}^{\prime }$, ${ℵ}_{2}\neq {ℵ}_{2}^{\prime }$ then WLOG say ${ℵ}_{2}<{ℵ}_{2}^{\prime }$ implication would be that $d(\zeta_{1},a_{1,1})\neq d(\zeta_{2},a_{1,1})$, because $d(\zeta_{1},a_{1,1})=d(\zeta_{2},a_{1,1})+t$ where $t={ℵ}_{2}^{\prime }-{ℵ}_{2}$ so $r(\zeta_{1}|R_{e_{2}})\neq r(\zeta_{2}|R_{e_{2}})$.

**Subcase 2:** If ${ℵ}_{1}\neq {ℵ}_{1}^{\prime }$, ${ℵ}_{2}={ℵ}_{2}^{\prime }$ then WLOG say ${ℵ}_{1}<{ℵ}_{1}^{\prime }$ implication would be that $d(\zeta_{1},a_{1,1})\neq d(\zeta_{2},a_{1,1})$, because $d(\zeta_{1},a_{1,1})=d(\zeta_{2},a_{1,1})+s$ where $s=2({ℵ}_{1}^{\prime }-{ℵ}_{1})$ so $r(\zeta_{1}|R_{e_{2}})\neq r(\zeta_{2}|R_{e_{2}})$.

**Subcase 3:** If ${ℵ}_{1}\neq {ℵ}_{1}^{\prime }$, ${ℵ}_{2}\neq {ℵ}_{2}^{\prime }$ then WLOG say ${ℵ}_{2}<{ℵ}_{2}^{\prime }$, ${ℵ}_{1}<{ℵ}_{1}^{\prime }$ implication would be that $d(\zeta_{1},R_{e_{2}})\neq d(\zeta_{2},R_{e_{2}})$, because $d(\zeta_{1},R_{e_{2}})=d(\zeta_{2},R_{e_{2}})+(s+t)$ so $r(\zeta_{1}|R_{e_{2}})\neq r(\zeta_{2}|R_{e_{2}})$.

**Case III:** When $\zeta_{1}=a_{{ℵ}_{1},{ℵ}_{2}}$ and $\zeta_{2}=b_{{ℵ}_{1}^{\prime },{ℵ}_{2}^{\prime }}$ then four more Subcases emerge.

**Subcase 1:** If ${ℵ}_{1}={ℵ}_{1}^{\prime }$, ${ℵ}_{2}={ℵ}_{2}^{\prime }$ implication would be that $d(\zeta_{1},a_{1,1})\neq d(\zeta_{2},a_{1,1})$, because $d(\zeta_{1},a_{1,1})$ least is comparable to $d(\zeta_{2},a_{1,1})+1$ so $r(\zeta_{1}|R_{e_{2}})\neq r(\zeta_{2}|R_{e_{2}})$.

**Subcase 2:** If ${ℵ}_{1}={ℵ}_{1}^{\prime }$, ${ℵ}_{2}\neq {ℵ}_{2}^{\prime }$ then WLOG say ${ℵ}_{2}<{ℵ}_{2}^{\prime }$ implication would be that $d(\zeta_{1},a_{1,1})\neq d(\zeta_{2},a_{1,1})$, because $d(\zeta_{1},a_{1,1})$ least is comparable to $d(\zeta_{2},a_{1,1})+2$ so $r(\zeta_{1}|R_{e_{2}})\neq r(\zeta_{2}|R_{e_{2}})$.

**Subcase 3:** If ${ℵ}_{1}\neq {ℵ}_{1}^{\prime }$, ${ℵ}_{2}={ℵ}_{2}^{\prime }$ then WLOG say ${ℵ}_{1}<{ℵ}_{1}^{\prime }$ implication would be that $d(\zeta_{1},a_{1,1})\neq d(\zeta_{2},a_{1,1})$, because $d(\zeta_{1},a_{1,1})$ least is comparable to $d(\zeta_{2},a_{1,1})+3$ so $r(\zeta_{1}|R_{e_{2}})\neq r(\zeta_{2}|R_{e_{2}})$.

**Subcase 4:** If ${ℵ}_{1}\neq {ℵ}_{1}^{\prime }$, ${ℵ}_{2}\neq {ℵ}_{2}^{\prime }$ then WLOG say ${ℵ}_{2}<{ℵ}_{2}^{\prime }$, ${ℵ}_{1}<{ℵ}_{1}^{\prime }$ implication would be that $d(\zeta_{1},a_{1,1})\neq d(\zeta_{2},a_{1,1})$, because $d(\zeta_{1},a_{1,1})$ least is comparable to $d(\zeta_{2},a_{1,1})+2$ so $r(\zeta_{1}|R_{e_{2}})\neq r(\zeta_{2}|R_{e_{2}})$.

When ${ℵ}_{1}\neq {ℵ}_{1}^{\prime }$, ${ℵ}_{2}\neq {ℵ}_{2}^{\prime }$ the positions of $\zeta_{1}$ and $\zeta_{2}$ where $d(\zeta_{1},a_{1,1})=d(\zeta_{2},b_{a,1})$, then $d(\zeta_{1},b_{v,1})\neq d(\zeta_{2},b_{v,1})$.

One can note from the Fig. 2 when $d(\zeta_{1},b_{1,1})=d(\zeta_{2},b_{1,1})$ implication would be that $d(\zeta_{2},b_{1,4h})\neq d(\zeta_{1},b_{1,4h})$ also $d(\zeta_{2},b_{v,1})=d(\zeta_{1},b_{v,1})$ implication would be that $d(\zeta_{1},b_{1,1})\neq d(\zeta_{2},b_{1,1})$.

we discuss all cases where $d(\zeta_{1},a_{1,1})\neq d(\zeta_{2},a_{1,1})$ while $d(\zeta_{1},b_{v,1})=d(\zeta_{2},b_{v,1})$

or $d(\zeta_{1},a_{1,1})=d(\zeta_{2},a_{1,1})$, $d(\zeta_{1},b_{v,1})\neq d(\zeta_{2},b_{v,1})$ From all the above cases, there is no possibility that the two representations are identical.

**Contrary Case**

contradiction the resolving set of cardinality one is possible if and only if it is a path graph, it contains a cycle and the minimum edge metric dimension 2 if a graph contains a cycle so the dimension 1 is not possible.

implies the resolving set has minimum cardinality two. Hence prove that $R_{e_{2}}$ is also the resolving set of nanosheet. □

**Figure 4:**
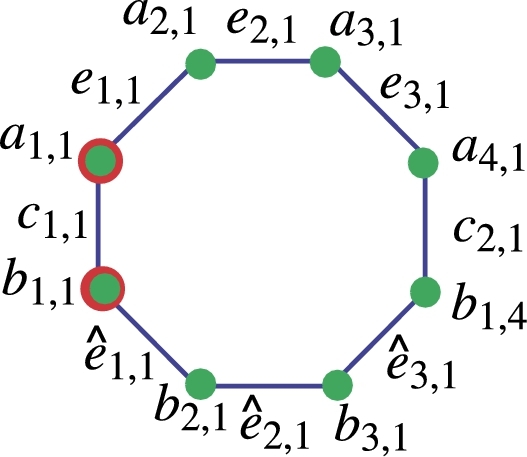
Octagone for sheet.

## 4 Exchange property

In finite-dimensional spaces, each vector is uniquely defined by elements constituting a vector space's basis, expressed through a linear combination. Similar to a linear basis in a vector space, the basis of a vector space is characterized by the exchange property. Likewise, each vertex in a finite graph can be uniquely identified through the vertices of a minimal resolving set. Consequently, resolving sets in finite graphs exhibit characteristics akin to bases in finite-dimensional vector spaces. However, unlike the linear basis of a vector space, the exchange property is not universally applicable to minimal resolving sets. Various studies in the literature have explored the presence of the exchange property in different graphs. For example, the exchange property holds for resolving sets in tree graphs, and it holds for determining sets but is false for resolving sets in wheel graphs for n≥8 [38].

Theorem 4.1 *The exchange property holds for an octagonal nanosheet*$NS_{h,v}$*if*h,v≥1*.*

To establish the exchange property, let's consider the minimal edge resolving set $R_{e_{1}}=a_{1,1},a_{1,4h}$ and another vertex $v=a_{v,1}$ belonging to $R_{e_{2}}$. According to the exchange property, $(R_{e_{1}}﹨u)\cup v$ should also form a minimal resolving set, where $u=a_{1,4h}$. Let's denote this set as *K*. Now, let's prove that *K* is indeed a minimal edge resolving set for $NS_{h,v}$. Since $R_{e_{1}}=a_{1,1},a_{1,4h}$, removing *u* from $R_{e_{1}}$ results in $R_{e_{1}}﹨u=a_{1,1}$. Adding vertex *v* to this set, we get $K=(R_{e_{1}}﹨u\cup v)=\{a_{1,1},b_{v,1}\}$. Now, by Theorem 3.3, we know that $R_{e_{2}}=a_{1,1},b_{v,1}$ is a minimal resolving set. Therefore, *K* is also a minimal resolving set. This demonstrates that the exchange property holds for $NS_{h,v}$ as the sets $R_{e_{1}}$ and $R_{e_{2}}$ can exchange the vertices *u* and *v* while maintaining the minimal edge resolving set property. The exchange property is valuable in various applications, such as network localization, defect diagnostics, and graph reconstruction. It facilitates the efficient creation and modification of resolving sets, enhancing the analysis and understanding of graph structures. It's important to note that not all resolving sets possess the exchange property, although it is beneficial when present.

**Conclusion**

This article explores two nanosheet structures derived from the octagonal grid. The minimal edge resolving sets for these nanosheets have been identified based on graph distances, and they have a cardinality of 2. Additionally, the study investigates the transformation of a nanosheet into a nanotube, observing that the conversion from 2D to 3D increases the cardinality of the edge resolving set by one. The analysis confirms the presence of the exchange property in this structural transformation.

### 4.1 Future study

We computed the exchange property and the single edge resolving set in this draught. It is also possible to talk about additional resolving set variations, such as fault-tolerant resolving sets [39], [40], [41], the chemical compounds' partition resolvability [42], [43], and other related theoretical parameters.

## CRediT authorship contribution statement

**Ali N.A. Koam:** Writing – review & editing, Data curation, Conceptualization. **Ali Ahmad:** Writing – review & editing, Funding acquisition, Formal analysis. **Sikander Ali:** Writing – original draft, Resources, Project administration. **Muhammad Kamran Jamil:** Writing – review & editing, Methodology, Investigation. **Muhammad Azeem:** Writing – review & editing, Visualization, Validation, Supervision.

## Declaration of Competing Interest

The authors declare that they have no known competing financial interests or personal relationships that could have appeared to influence the work reported in this paper.

## Data Availability

No data was used for the research described in the article
